# The effects of background noise and behavioral context on the acoustic characteristics of coo calls in Japanese macaques

**DOI:** 10.1007/s10329-025-01213-4

**Published:** 2025-09-11

**Authors:** Noriko Katsu, Kazuo Inami, Kazunori Yamada

**Affiliations:** Graduate School of Human Sciences, The University of Osaka, 1-2 Yamadaoka, Suita, Osaka Japan

**Keywords:** Vocal plasticity, Anthropogenic noise, Vocal communication, contact call, Primate

## Abstract

Anthropogenic noise may affect the spectral and temporal structures of contact calls in wild non-human primates. However, most studies have compared groups living at varying distances from noise sources, while few have focused on the relationship between specific noise types and vocalizations at the individual level, which is necessary to examine vocal plasticity. We aimed to clarify the effects of background noise on the acoustic characteristics of coo calls of Japanese macaques on an individual basis; thus, we examined the effects of noise levels, behavioral context, and number of individuals in proximity on the duration and spectral measure of calls. We conducted focal observations of adult female Japanese macaques, and recorded coo calls and background noise levels. We analyzed 128 calls from nine subjects and found that the start and mean frequencies of coo calls were higher at higher noise levels. The call duration and frequency modulations were higher when the number of individuals in proximity was lower and the end frequency was higher during foraging and moving. These findings suggest that different factors affect the acoustic characteristics of the coo calls of Japanese macaques simultaneously. The most intense background noise sources were typically low-frequency sources that overlapped the frequency bands of the calls. Hence, they exerted a profound influence on call acoustics, which is consistent with the results of previous studies. Within-individual adjustments to ambient noise indicate vocal plasticity, enabling efficient signal transmission to the receiver.

**Supplementary Information:**

The online version contains supplementary material available at 10.1007/s10329-025-01213-4.

## Introduction

Ambient noise is considered a strong selective pressure in animal acoustic communication (Brumm and Slabbekoorn 2005). By analyzing how nonhuman primates adjust their acoustic characteristics in response to noise, we can gauge the extent of vocal plasticity based on auditory feedback, which was previously considered to be very limited in nonhuman primates (Hammerschmidt and Fischer 2008). An increase in vocalization amplitude in response to increased noise is known as the Lombard effect (Hotchkin and Parks 2013). This has been observed in various taxa of fish, birds, and mammals, indicating a common subcortical mechanism among these vertebrate species (Kunc et al. 2022). Modifications other than amplitudes, such as the spectral and temporal structures of vocalization, have also been reported in birds and mammals (Brumm and Slabbekoorn 2005). For example, changes in the duration of calls can increase the signal detection time for receivers (Bee and Micheyl 2008), and a shift in the spectrum can reduce masking by noise (Francis et al. 2011).

The effect of noise on contact calls in non-human primates has been investigated in both captive and natural environments. In captive settings, two macaque species (*Macaca fascicularis* and *M. nemestrina*) showed increased levels of call amplitude when presented with low-frequency band noise (200–500 Hz) that masks the fundamental frequency of their calls (Sinnott et al. 1975). However, this change was not observed when presented with high-frequency band noise (8–16 kHz; Sinnott et al. 1975). The amplitude of calls was also increased by playback of white noise in common marmosets (*Callithrix jacchus*; Brumm et al. 2004) and cotton-top tamarins (*Saguinus oedipus*, Roian Egnor and Hauser 2006). Increased syllable duration often coincides with an increase in amplitude (Brumm et al. 2004; Hotchkin et al. 2013; Roian Egnor and Hauser 2006). Regarding spectral modifications, studies on cotton-top tamarins have reported an increase in peak frequency with increasing noise levels and a decrease in minimum frequency in response to broader band noise (Hotchkin et al. 2015).

In wild settings, black-tufted marmosets (*Callithrix penicillata*) show longer and lower calls at the site near a mining site (Bittencourt et al. 2023; Santos et al. 2017), probably because these low-frequency calls are less attenuated and travel farther than higher-frequency calls (Mitani and Stuht 1998). Black-fronted titis (*Callicebus nigrifrons*) living near a mining site emitted shorter calls, unlike those in most previous studies, probably because of the increased chance of avoiding masking by intermittent noise (Duarte et al. 2018). Sobroza et al. (2024) reported that pied tamarins (*Saguinus bicolor*) exhibited a reduced syllable repetition rate for long calls when noise levels were increased. Previous studies conducted in wild settings have shown that the modification of vocalization varies depending on noise and species characteristics. These findings indicate the need to analyze the corresponding noise types and call utterances. However, most studies conducted in wild environments compared groups living in areas near and far from noise sources. Few studies in wild environments have investigated vocal modifications on an individual or group basis (Santos et al. 2017; Sobroza et al. 2024). To clarify vocal plasticity, that is, the ability to modify call characteristics in response to increased acoustic inference by noise, it is necessary to examine within-individual vocal modifications based on changes in noise levels.

Moreover, few studies have reported the effect of behavioral context in conjunction with the effect of noise on vocal modification. Sobroza et al. (2024) reported the effects of group size and distance from the home range border on the occurrence and frequency of long calls in pied tamarins. However, the temporal and spectral features of calls can also be affected by other environmental and motivational factors. Proximity to group members (Sugiura 2007b) can affect vocal characteristics related to the transmission of signals to other individuals. Low visibility of the environment, which increases separation risk, increases call rates (Koda et al. 2008) and causes call modifications (Candiotti et al. 2012). Motivational factors such as activity can also affect the spectral features of calls (Dittus 1984; Hauser 1991; Owren and Casale 1994). To date, no study has examined the effects of noise on vocal modifications by considering other contextual factors.

Coo calls are contact calls of macaques, including Japanese macaques, and have graded characteristics (Green 1975). The function of coo calls is to maintain spatial cohesion among group members (Koda et al. 2008; Sugiura 2007b). Modifications and changes in coo calls in a behavioral context have been relatively well investigated in Japanese macaques. Sugiura (2007b) showed that call duration and frequency modulation increase as the distance between group members increases. Green (1975) categorized coo calls into seven subtypes according to their frequency patterns and showed that the occurrence rate of each subtype varied based on the situation, but gradually overlapped. However, a subsequent study investigated whether the two subtypes of coo calls differ in the peak position of the fundamental frequency (smooth early high and smooth late high) proposed by Green, and found that the peak position of coo calls was not categorized into these two distinct types (Owren and Casale 1994). Situational differences in coo calls have been studied, particularly in food-related situations. Coo calls are often emitted during foraging (Koda et al. 2008; Suzuki and Sugiura 2011), and are considered food-associated calls (Koda 2012). In rhesus macaques, food-associated coo calls have specific features such as broad bandwidth (Hauser 1991). In summary, coo calls are good targets for investigating noise-induced vocal modifications, as their acoustic characteristics can be modified according to the context to enhance call transmission effectiveness. Although there is no consensus on how activity affects vocal modification, it needs to be considered when examining the effects of background noise.

This study aimed to clarify the effects of background noise on the acoustic characteristics of the coo calls of Japanese macaques on an individual basis. If macaques modulate their calls flexibly to communicate efficiently, we predict that call duration would be longer in noisy environments. We also expected that noise overlapping with the frequency band of coo calls would affect call characteristics more. Specifically, the spectral features would change such that calls would avoid the frequency bands of the noise. We observed Japanese macaques of the Arashiyama group (Kyoto, Japan), which were exposed to both low-frequency anthropogenic noise (human speech sounds and mechanical noise) and high-frequency environmental noise (sounds of insects such as *Cicadoidea*). We examined the effects of activity and proximity of other individuals, together with the effects of noise.

## Methods

### Study site and subjects

This study was conducted on a free-ranging group of Japanese macaques at Arashiyama Monkey Park (35°00′N and 135°67′E), Kyoto, Japan. Observational research on the Arashiyama group based on individual identification has been conducted since the 1950s, and maternal kinship patterns have been recorded to date (Fedigan and Asquith 1991). The park staff fed the group four times daily with wheat grain and soybeans at the provisioning ground and once near the sleeping site. During the opening time (09:00–16:30), the monkeys remained within a radius of approximately 300 m from the provisioning ground (approximately 100 m^2^). At the beginning of the study period, this group included 136 individuals. The focal subjects were 11 adult females, with an average age of 18.1 ± 8.4 SD years old (range: 8–31; Table 1). We solely observed females, as female macaques make coo calls far more frequently than males (Greeno and Semple 2009). The subjects were selected based on the dominance rank of their matrilineal lineage, age, and kinship to avoid bias. All subjects were maternally unrelated (more distant than cousins).

**Table 1:** Summary of coo calls recorded and analyzed based on the activity of nine subjects

Subject ID	Age	# of focal sessions (min)^a^	# of calls analyzed (*N* = 128)	# of calls recorded	Activity (*N* = 128)		
					Social interaction	Resting	Foraging and moving
Ch'15	8	12 (200)	18	18	5	4	9
Co'90′01	22	18 (300)	11	14	3	8	-
Co'14	9	14 (259)	13	14	2	8	3
Co'13	10	16 (276)	13	22	4	9	-
Ku'01	22	16 (205)	19	19	9	6	4
Mi'95	28	19 (335)	10	11	2	6	2
Mo'11	12	17 (241)	20	22	4	10	6
Ra'15	8	17 (241)	8	8	1	2	5
Yu'96	27	13 (251)	16	17	2	11	3

Two subjects (aged 31 and 22 years) for whom two or fewer calls were recorded were excluded from the analysis; thus, they do not appear in the table

^a^Including the focal sessions that were finished before 20 min had elapsed

### Vocal and behavioral data

**Fig. 1:**
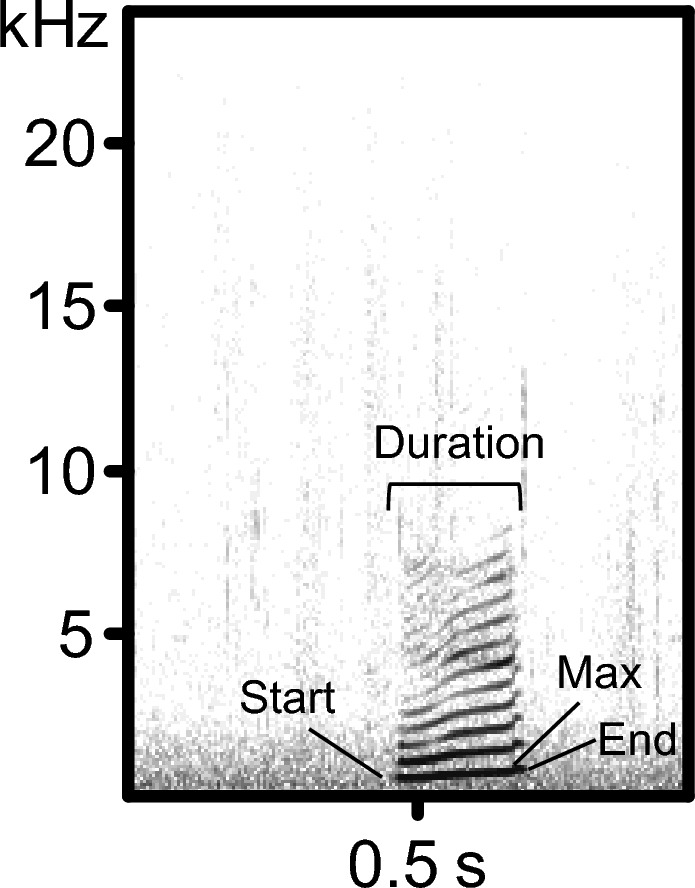
The example of a coo call emitted when there was no specific background noise. Solid lines indicate the start, max, and end fundamental frequency

Data were collected between July and November 2023. Behavioral observations were conducted between 09:00 and 16:30, except 15 min after provisioning. The observation area includes an area for visitors and an area close to them. The subjects were observed in a predetermined randomized order. The observer (KI) conducted 20-min focal observations and recorded coo calls using a directional microphone (RODE NTG2, Sydney, Australia) and a digital audio recorder (OLYMPUS V802/803, Tokyo, Japan), with a sampling rate of 44.1 kHz and 16-bit resolution. Coo calls (Fig. 1) are defined as vocalizations with a tonal structure and a variable fundamental frequency component (Green 1975). When the focal subject emitted a call, the subject’s activity and the number of audiences, individuals within 5 m of the subject were recorded by direct observation. The activities were classified as resting, social interaction, moving, and foraging. The observer maintained a distance of 2–5 m from the focal subject during the observation, directing the microphone at the face of the focal subject. A total of 2759 min (131–335 min per subject) of focal observations were recorded. We recorded 148 calls (13.5 ± 7.3 calls per subject on average), but we excluded two subjects because only one or two calls were recorded from them (Table 1).

We excluded 20 calls that were not of good quality; thus, analyses were conducted on 128 calls (14.2 ± 4.2 calls per subject on average) from nine subjects. Spectrograms were generated by fast Fourier transformation (FFT length = 512, overlap = 50%, sampling frequency = 22.05 kHz, with a Hamming window) using SASLab Pro software (Avisoft Bioacoustics, Glienicke, Germany). Acoustic characteristics were calculated using “automatic parameter measurement” within the software to an accuracy of 10 ms and 100 Hz and inspected visually. Seven acoustic parameters were measured and used for the analyses: call duration (s); start, end, maximum, and mean fundamental frequency (F0); and modulation of F0. The modulation of F0 is the difference between the maximum and the start or end frequencies. We did not use the amplitude measure of the calls for the analyses because the distance between the subject and the observer varied.

### Noise level data

Noise levels were continuously recorded during focal behavioral observations using a sound meter logger (CUSTOM SL-1373SD; Tokyo, Japan). The noise meter was carried by the observer and fixed to the observer's torso with the microphone facing upward. Background noise levels (dB) were instantaneously sampled every 5 s during observation, with an A-weighted frequency response and Fast time constant. The noise levels before and 1 min after the beginning of the call utterance were averaged and used for analysis as the noise level at the point of call utterance. This procedure was performed to reduce the effect of vocalization itself on the noise level. Visual inspection revealed that the background noise sources were sounds from insects (*n* = 32, such as *Cicadoidea,* and *Gryllidae*), machines or vehicles (*n* = 21), and human voices (*n* = 18). No noise was detected in the remaining 57 calls.

### Statistics

To examine the factors affecting the acoustic parameters of coo calls, we used linear mixed models (LMMs). Six acoustic parameters were set as response variables (Table 2) and a model was tested for each variable. The fixed effects were the same in all models: noise level, audience size, and activity (resting, social interactions, moving, and foraging) during the call utterances. Calls made while moving and foraging were grouped together in the analysis, because moving often occurs in conjunction with foraging. In addition, an interaction effect between noise level and audience size was included in each model to test whether the vocal modifications in response to the noise levels occur only when an audience is nearby, although call duration or modulation is generally expected to increase when there is no audience nearby (i.e., total number of audiences is low). The subject ID was entered as a random effect in each model. The noise level and audience were centralized in the models. All the tests were conducted using R version 4.2.3 (R Core Team 2023). The LMMs were performed using lme4 (Bates et al. 2013) and emmeans packages.

**Table 2:** The descriptive statistics of acoustic parameters used in the analyses (*N* = 128 from nine subjects)

Parameter	Mean ± SD
Duration (s)	0.284 ± 0.067
F0 start (Hz)	418 ± 110
F0 end (Hz)	415 ± 103
F0 max (Hz)	560 ± 101
F0 mean (Hz)	623 ± 193
F0 modulation (Hz)	418 ± 65

## Results

**Fig. 2:**
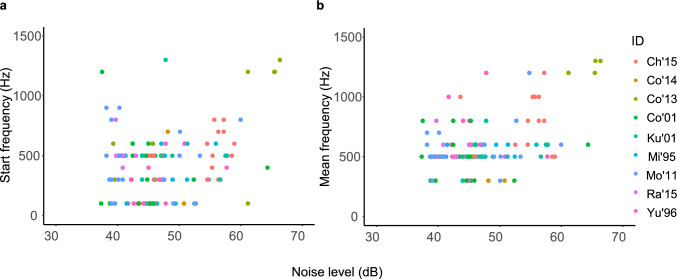
The effect of background noise levels on the **a** start and **b** mean fundamental frequencies of the coo calls (*N* = 9)

**Fig. 3:**
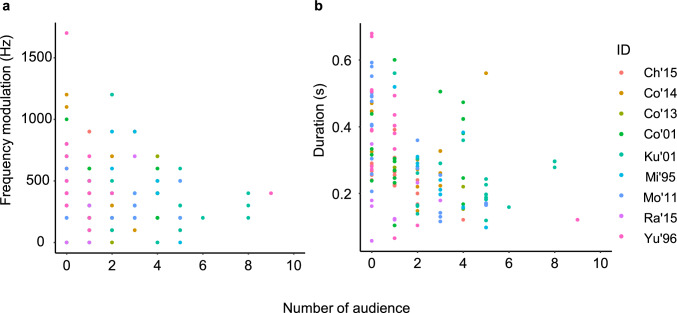
The effect of number of nearby individuals on the **a** amount of frequency modulation and **b** call duration of the coo calls (*N* = 9)

**Fig. 4:**
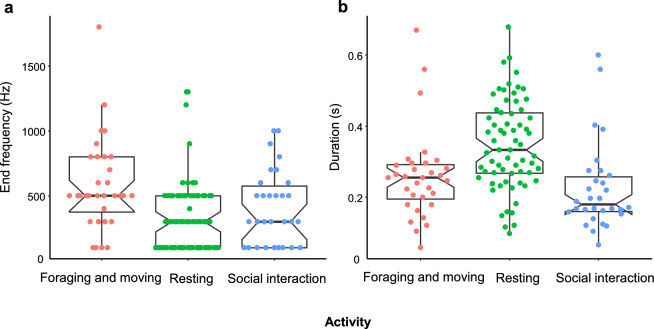
The end frequency **a** and duration **b** based on activity: resting, foraging, moving, and social interaction (*N* = 9)

Noise levels ranged from 38.1 to 61.3 dB (mean ± SD: 47.2 ± 5.4) at the time of coo calls. The descriptive statistics of the acoustic parameters of the coo calls are summarized in Table 2. The background noise level had a significant effect on the start and fundamental frequencies of the coo calls (Table 3). The start and mean frequencies of coo calls increased at high noise levels (Fig. 2a–b). Noise levels did not significantly affect the call duration or frequency modulation (Table 3; Fig. S1). The number of audience had a significant effect: when there were few individuals in close proximity, the call duration was longer and the frequency modulation was larger (Fig. 3a–b). The interaction between noise level and number of audience was not significant in any models. Activity significantly affected end frequency and call duration. Multiple comparisons revealed that the end frequency of coo calls during foraging and moving was higher than during resting (*p* = 0.003; Fig. 4a; Table S1). The difference between other activities was not significant (foraging-moving vs. social interaction: *p* = 0.110; resting vs. social interactions: *p* = 0.533). Call durations were longer during resting than during social interactions (*p* = 0.005; Fig. 4b; Table S1), and marginally longer than during foraging and moving (*p* = 0.081). Thus, the noise level, proximity to other individuals, and activity affected different parameters.

**Table 3:** The results of linear mixed models (LMMs) for the effect of noise level, number of individuals in proximity, and activity on the acoustic parameters

Explanatory variables	Fixed effect	Estimate	SE	df	t	*p*
Duration(s)						
	Intercept	0.267	0.026	24	10.072	0.000
	Noise level (dB)	−0.001	0.002	116	−0.544	0.587
	Audience	**−0.021**	**0.006**	**115**	**−3.357**	0.001***
	Activity: social interaction	**−**0.028	0.031	122	**−**0.913	0.363
	Activity: resting	**0.060**	**0.027**	**122**	**2.205**	0.029*
	Noise level (dB)*Audience	0.001	0.001	114	1.022	0.309
F0 start (Hz)						
	Intercept	402.620	54.910	31	7.332	0.000
	Noise level (dB)	**14.320**	**4.210**	**117**	**3.402**	0.001***
	Audience	**−**18.870	14.350	116	**−**1.315	0.191
	Activity: social interaction	32.840	69.800	122	0.470	0.639
	Activity: resting	14.050	62.848	119	0.230	0.819
	Noise level (dB)*Audience	**−**3.280	2.470	98	**−**1.328	0.187
F0 end (Hz)						
	Intercept	583.259	54.005	122	10.800	0.000
	Noise level (dB)	2.536	4.807	122	0.528	0.599
	Audience	**−**9.706	16.417	122	**−**0.591	0.555
	Activity: social interaction	**−162.668**	**77.800**	**122**	**−2.091**	0.039*
	Activity: resting	**−239.047**	**66.524**	**122**	**−3.593**	0.000***
	Noise level (dB)*Audience	**−**2.626	2.586	122	**−**1.015	0.312
F0 max (Hz)						
	Intercept	637.538	48.982	38	13.016	0.117
	Noise level (dB)	5.317	4.120	119	1.291	0.199
	Audience	−19.330	14.057	118	−1.375	0.172
	Activity: grooming	−93.378	67.454	120	−1.384	0.169
	Activity: rest	−86.589	58.396	105	−1.483	0.141
	Noise level (dB)*Audience	−1.668	2.311	70	−0.722	0.473
F0 mean (Hz)						
	Intercept	619.316	81.968	14	7.556	0.000
	Noise level (dB)	**9.212**	**4.191**	**115**	**2.198**	0.030*
	Audience	−21.725	14.249	114	−1.525	0.130
	Activity: social interaction	−27.880	71.035	119	−0.392	0.695
	Activity: resting	35.081	62.739	119	0.559	0.577
	Noise level (dB)*Audience	3.680	2.615	121	1.407	0.162
F0 modulation (Hz)						
	Intercept	384.605	43.727	122	8.796	0.000
	Noise level (dB)	1.015	3.892	122	0.261	0.795
	Audience	**−26.781**	**13.292**	**122**	**−2.015**	0.046*
	Activity: social interaction	−49.879	62.993	122	−0.792	0.430
	Activity: resting	28.287	53.863	122	0.525	0.600
	Noise level (dB)*Audience	−1.870	2.094	122	−0.893	0.374

## Discussion

This study examined the effects of background noise, number of audiences, and activity on the acoustic characteristics of coo calls. We found that the noise levels affected the acoustic characteristics, particularly the spectral features of the fundamental frequency. We also found that the number of audience and activities affected different characteristics of noise levels. Here, we discuss the possibility of acoustic modifications caused by each factor.

In our study, the effect of noise level was only observed at the start and mean frequencies. However, the modification in frequency may not be determined simply by the noise levels; the noise level and frequency band may have exerted confounding effects. Noise levels and frequency bands were strongly associated with our observations; louder noise levels were recorded when low background noise (around 1 kHz), such as speech sounds from visitors or machine noises, were predominant (54.9 ± 5.4 dB) than when high background noises (approximately 4–10 kHz) such as insect sounds predominated (44.8 ± 3.6 dB). The former sound sources were relatively similar to or sometimes overlapped with the coo calls, in contrast to the latter. In such an environment, higher-frequency coo calls would be transmitted more effectively and avoid overlapping with low-frequency noise. The increase in the start frequency appeared to be particularly noticeable when the noise exceeded 50 dB (Fig. 2). However, the call amplitude and frequency can increase simultaneously. Although we speculate that the noise frequency band strongly explains the modifications in call frequency, it is not possible to make a definite judgment based on the current data. Therefore, further studies in different noise environments are required. Noise-induced spectral modifications in vocalization have been reported in captive tamarins when white noise was played (Hotchkin et al. 2015) and in wild marmosets in the presence of mining noise (Bittencourt et al. 2023; Santos et al. 2017). Our study is consistent with these studies in that it shows that noise with a frequency that overlaps with vocalizations can affect their spectral features.

Noise levels did not affect call duration, although call duration reportedly changes with background noise levels (Brumm et al. 2004; Hotchkin et al. 2013; Macpherson and Middlebrooks 2000; Roian Egnor and Hauser 2006). This discrepancy between the current and previous studies may be due to the differences in noise environments. Many studies on wild primates have focused on artificial and loud noises, such as mining and traffic noises (Bittencourt et al. 2023; Duarte et al. 2018). The noise at our study site was primarily due to human speech and insect calls, and might be moderate compared to that in previous studies. The frequency bands of these noises are relatively narrow; thus, avoiding frequency overlap may enable efficient call transmission, resulting in relatively fewer effects on call duration. However, the cost of modifying spectral features may differ from that of durational modifications, as relatively few studies have reported spectral changes (Hotchkin et al. 2015; Santos et al. 2017) in contrast to temporal changes (Brumm et al. 2004; Macpherson and Middlebrooks 2000) in primates. How the modification in response to noise changes according to cost and efficiency should be considered.

Our study showed that the number of individuals within 5 m of the caller affected call duration and frequency modulation. There was no significant interaction between the number of audience and the noise level, indicating that these two factors have a certain degree of independent influence on call modification. Coo calls are used to distant individuals to maintain group cohesion (Koda et al. 2008; Sugiura 2007a) as well as in social interactions with nearby individuals (Katsu et al. 2016). Longer calls offer distant receivers greater detectability and locatability. As our study site was a provisioned group with relatively close inter-individual distances, we could not use the same definition used in a previous study (Sugiura 2007b); wild Japanese macaques emit coo calls with longer durations and greater frequency modulation when no individuals are within a 10 m radius. Both duration and frequency modulation are important for locatability; for example, longer and more modulated calls can enhance interaural phase differences, making it easier to locate a sound source (Snowdon and Hodun 1981).

The modification of call features can be interpreted as an audience effect, that is, changes in signaling behaviors due to the presence of others (Zuberbuhler 2008). The audience effect has been shown in alarm or food calls among nonhuman primates, suggesting that primates use vocalizations in a goal-directed manner. By suppressing or enhancing vocal behaviors, they can control the information gained by receivers and increase the benefit to the caller (Schamberg et al. 2018). Coo calls may also benefit callers if the information reaches individuals who have strong bonds with them. For example, chimpanzees increase their long-distance pant hoots when closely bonded individuals are out of sight (Schamberg et al. 2018). Although our study did not record the composition of the audience or the responses from potential recipients, a similar trend may exist for Japanese macaques. In any case, our results indicate that coo calls are adjusted to communicate with potential receivers, although changes may also reflect the caller’s arousal state (Owren et al. 2011).

Food-associated calls in macaques are characterized by acoustic features such as greater bandwidth (Hauser 1991) or frequency modulation (Dittus 1984), although food-associated coo calls cannot be discriminated based solely on their acoustic features (Hauser 1991; Hauser and Marler 1993). The end fundamental frequency of coo calls during foraging and moving was significantly higher than that during rest. This characteristic likely reflects the food-associated calls in this group. Monkeys in this group often vocalize coo calls with a high frequency at the end when feeding is imminent. Food-associated calls can be shaped relatively flexibly in specific contexts (Hihara et al. 2003). Considering that the acoustic characteristics do not exactly match those in previous studies (Dittus 1984; Hauser 1991), they may reflect modifications through interactions with humans rather than a more general tendency of food-associated calls. Calls made during rest were longer than those made during social interactions; additionally, they tended to be longer than those made during foraging and moving. This result is consistent with the report that coo calls are generally less likely to be targeted at specific individuals compared with other short-distance affiliative calls, which include short low coos (i.e., coo calls shorter than 0.2 s, Katsu et al. 2016). Previous studies have found no clear differences in variation among contexts, particularly calls made during resting; however, calls made during social interactions were not included in these studies (Owren and Casale 1994; Sugiura 2007b). The characteristics of coo calls might differ under very limited and specific contexts, such as social interactions, although the activity itself does not exert a significant effect in general.

We averaged the noise levels before and after 1 min and recorded them every 5 s. We could not exclude the call amplitude itself from the noise levels because the noise meter and recording device for vocalization were not completely synchronized in terms of time, so the 5-s sampling point that could contain the call could not be accurately extracted. However, we believe that the evaluation of noise level did not change significantly due to the call itself. The sound level meter was pointed upwards and not directly toward the caller. The average noise levels measured 1 min before and after call utterances were highly correlated with the average noise levels during the focal session (20 min, Pearson correlation coefficient, *N* = 128, *r* = 0.829). This suggests that the noise level during call utterances accurately reflects the overall noise environment for a given period. Moreover, previous studies have reported the increase in call amplitude was reported to be about 2 dB for every 10 dB in macaques (Sinnott et al. 1975), and within 5–10 dB for every 10 dB in marmosets (Brumm, et al. 2004). Considering that other temporary sounds may also have been included, the variation in noise levels induced by call amplitude was not considered significant. Regardless, the recorded background noise level may have changed depending on the distance from the caller. Future studies should address the problem of measuring call sound pressure.

Individual differences may also have affected the variations in acoustic characteristics. Thus, the effects of noise levels or behavioral contexts could have been caused by individual differences, although we have controlled for individual ID as random effects in the models. The number of calls recorded varied among the individuals, and some subjects lacked call samples for specific activities or proximity situations. However, previous studies have revealed that individual differences are more pronounced in formants created by individual vocal tract characteristics in some primates, such as humans and baboons (Bachorowski and Owren 1999; Owren et al. 1997), and that macaques use formants as cues for individual identification rather than the pitch of the fundamental frequency (Furuyama et al. 2016). Although some subjects (Ch15 and Co14) showed relatively higher fundamental frequency measures than others, they overlapped among subjects. Because the effect of individual differences cannot be completely ruled out, more calls from various individuals should be examined in the future.

In conclusion, background noise and behavioral context had different effects on the different characteristics of coo calls in Japanese macaques. Spectral changes in response to noise suggest that plasticity may be used to avoid noises overlapping with call frequencies. These findings suggest that different factors simultaneously affect macaque call utterances, such as auditory feedback in noisy environments and motivational changes according to activity or proximity to group members. The ability to adaptively modify the spectral or durational components of calls ensures vocal transmission between individuals in various environmental and social situations.

## Supplementary Information

Below is the link to the electronic supplementary material.

Supplementary file1 (DOCX 49 KB)

## Data Availability

The data are included in the supplementary materials.
