# Supplementary material for: The effects of background noise and behavioral context on the acoustic characteristics of coo calls in Japanese macaques
Source: Primates. 2025 Sep 11;66(6):545–54. doi: 10.1007/s10329-025-01213-4 (PMC12680763; doi:10.1007/s10329-025-01213-4)
Supplement: Supplementary file 1 — Supplementary file1 (DOCX 49 KB) [file 10329_2025_1213_MOESM1_ESM.docx]

**The effects of background noise and behavioral context on the acoustic characteristics of coo calls in Japanese macaques**

***Primates***

**Noriko Katsu^1*†^, Kazuo Inami^1†^, Kazunori Yamada^1^**

^1^ Graduate School of Human Sciences, Osaka University, Suita, Osaka, Japan

**^†^**These authors equally contributed to this work and share first authorship.

***Corresponding author**: Noriko Katsu, Graduate School of Human Sciences, Osaka University, 1-2 Yamadaoka, Suita, Osaka, Japan. [n.katsu.hus@osaka-u.ac.jp](mailto:n.katsu.hus@osaka-u.ac.jp)

**Fig. S1** The effect of background noise levels on the (a) frequency modulation and (b) call duration of the coo calls (N = 9)

**Table S1** Multiple comparison of the end frequency and call duration of coo calls based on activity.

| Explanatory variables | Level | Estimate ± SE | p |
| --- | --- | --- | --- |
| F0 end (Hz) | |  |  |
|  | Foraging and moving-Resting | 230.0±69.8 | 0.004 |
|  | Foraging and moving-Social interaction | 159.1±79.9 | 0.119 |
|  | Resting-Social interaction | -70.9±70.9 | 0.579 |
| Duration (s) | |  |  |
|  | Foraging and moving-Resting | -0.060±0.028 | 0.081 |
|  | Foraging and moving-Social interaction | 0.028±0.031 | 0.640 |
|  | Resting-Social interaction | 0.089±0.028 | 0.005 |
|  |  |  |  |
